# The effect of surgery on fat mass, lipid and glucose metabolism in mild primary hyperparathyroidism

**DOI:** 10.1530/EC-18-0259

**Published:** 2018-07-16

**Authors:** Kristin Godang, Karolina Lundstam, Charlotte Mollerup, Stine Lyngvi Fougner, Ylva Pernow, Jörgen Nordenström, Thord Rosén, Svante Jansson, Mikael Hellström, Jens Bollerslev, Ansgar Heck, the SIPH Study Group

**Affiliations:** 1 Section of Specialized EndocrinologyOslo University Hospital, Oslo, Norway; 2 Department of RadiologyInstitute of Clinical Sciences, Sahlgrenska Academy at the University of Gothenburg, Sahlgrenska University Hospital, Gothenburg, Sweden; 3 Clinic of Breast and Endocrine SurgeryCenter HOC, Copenhagen University Hospital, Rigshospitalet, Copenhagen, Denmark; 4 Department of EndocrinologySt. Olavs Hospital, Trondheim, Norway; 5 Departments of Molecular MedicineSurgery and Endocrinology, Karolinska Institutet, Karolinska University Hospital, Stockholm, Sweden; 6 Department of Molecular Medicine and SurgeryKarolinska Institutet, Stockholm, Sweden; 7 Department of MedicineSahlgrenska University Hospital, Gothenburg, Sweden; 8 Department of Endocrine SurgerySahlgrenska University Hospital, Gothenburg, Sweden; 9 Faculty of MedicineUniversity of Oslo, Oslo, Norway

**Keywords:** primary hyperparathyroidism, lipids, glucose homeostasis, DXA and body composition

## Abstract

**Context:**

Mild primary hyperparathyroidism has been associated with increased body fat mass and unfavorable cardiovascular risk factors.

**Objective:**

To assess the effect of parathyroidectomy on fat mass, glucose and lipid metabolism.

**Design, patients, interventions, main outcome measures:**

119 patients previously randomized to observation (OBS; *n* = 58) or parathyroidectomy (PTX; *n* = 61) within the Scandinavian Investigation of Primary Hyperparathyroidism (SIPH) trial, an open randomized multicenter study, were included. Main outcome measures for this study were the differences in fat mass, markers for lipid and glucose metabolism between OBS and PTX 5 years after randomization.

**Results:**

In the OBS group, total cholesterol (Total-C) decreased from mean 5.9 (±1.1) to 5.6 (±1.0) mmol/L (*P* = 0.037) and LDL cholesterol (LDL-C) decreased from 3.7 (±1.0) to 3.3 (±0.9) mmol/L (*P* = 0.010). In the PTX group, the Total-C and LDL-C remained unchanged resulting in a significant between-group difference over time (*P* = 0.013 and *P* = 0.026, respectively). This difference was driven by patients who started with lipid-lowering medication during the study period (OBS: 5; PTX: 1). There was an increase in trunk fat mass in the OBS group, but no between-group differences over time. Mean 25(OH) vitamin D increased in the PTX group (*P* < 0.001), but did not change in the OBS group. No difference in parameters of glucose metabolism was detected.

**Conclusion:**

In mild PHPT, the measured metabolic and cardiovascular risk factors were not modified by PTX. Observation seems safe and cardiovascular risk reduction should not be regarded as a separate indication for parathyroidectomy based on the results from this study.

## Introduction

Primary hyperparathyroidism (PHPT) is a common endocrine disease (1) and is characterized by hyperactivity in one or several parathyroid glands, raised levels of parathyroid hormone (PTH) and altered calcium homeostasis with increased calcium levels in the circulation. Mild PHPT is the predominant clinical phenotype in the western world. It is characterized by biochemical evidence of PHPT and absence of typical target organ involvement, such as nephrolithiasis and skeletal disease. Treatment in overt PHPT is parathyroidectomy while the optimal management of mild or asymptomatic PHPT has been discussed for a long time. Observational studies have demonstrated an association of increased calcium levels and PHPT to cardiovascular disease (2, 3). PTH is a metabolically active hormone and elevated levels of PTH are associated with alterations in cardiac structure and function, cardiovascular risk factors and overweight (4, 5, 6, 7). Some studies, but not all, have demonstrated improvement of cardiovascular disease or risk factors after surgery (3, 8, 9).

Furthermore, it has been demonstrated that PHPT patients with diabetes or dyslipidemia can improve their dysmetabolic status after parathyroidectomy (10, 11). On the basis of these findings, it has been proposed to include cardiovascular risk among the indications for parathyroidectomy (12).

Nevertheless, evidence for the effect of surgery on cardiovascular disease or cardiovascular risk factors is weak as many of these studies were of short term and observational by design. There may also be a publication bias toward studies with positive results. Thus, in the absence of strong evidence, surgery should not be considered to improve cardiovascular risk markers according to the guidelines from most recent international workshop on PHPT (13, 14).

PTH levels are closely related to body weight, BMI and vitamin D status. The causality behind these associations is not completely understood (7, 15). It has been suggested that PTH may lead to weight gain by inhibition of lipolysis (16) and thereby promote insulin resistance (15). Thereby, PHPT is related to manifestations of the metabolic syndrome, and potential effects on body composition and insulin resistance may emerge long time also in mild disease. To our knowledge, there are no data assessing the long-term effect of surgery on body mass and fat distribution in mild PHPT. Longitudinal assessment of body composition with dual energy X-ray absorptiometry (DXA) is a reliable tool to assess changes in body composition (17).

The Scandinavian Investigation on Primary Hyperparathyroidism (SIPH) was a prospective, randomized and controlled trial in patients with mild and asymptomatic PHPT. The patients were randomized to either parathyroidectomy (PTX) or observation without intervention (OBS). We found no significant treatment effect of PTX on cardiovascular risk factors including serum lipids after 2 years of follow-up in the SIPH study (9).

The aim of the present study was to evaluate treatment effects of PTX compared with OBS on body composition, serum lipids and glucose homeostasis, after 5 years. Our hypothesis was that PTX would have a beneficial effect on fat mass, lipids and glucose metabolism in mild PHPT.

## Materials and methods

### Subjects

From 1999 to the end of inclusion by June 2005, the SIPH study (ClinicalTrials.gov: Nbib522028) included a total of 191 patients (26 men) with asymptomatic (mild) PHPT, as previously described in detail (18). Key inclusion criteria were untreated mild PHPT, albumin-corrected serum calcium level between 2.60 and 2.80 mmol/L and age between 50 and 80 years. Key exclusion criteria were hyperparathyroid bone disease, serum creatinine >130 µmol/L and kidney stones. At inclusion, 95 patients were randomized to OBS, and 96 to PTX. Inclusion and exclusion criteria and primary and secondary effect parameters have previously been described in detail (19). According to the protocol, samples were drawn and analyzed at given time points at baseline, after 2, 5 and 10 years. A total of 145 patients were still in the protocol after 5 years (19). The present paper includes 119 patients (103 female, 16 male) who had performed the 5-year visit and had frozen blood samples and/or DXA available from the baseline visit and the 5-year follow-up.

### Ethics

Written informed consent was obtained from all participants, and the study was conducted according to the Declaration of Helsinki II. The study was approved by the independent ethical committees in Denmark (Århus Amt), Norway (Regional Ethical Committee Health Region South-East) and Sweden (Ethical Committees Gothenburg, Stockholm and Uppsala).

### Biochemical analyses

At the baseline and 5-year visit, blood samples were drawn after an overnight fast. The biochemical analyses serum/plasma calcium, albumin and PTH were run consecutively locally by standard laboratory procedures and have been described previously (19). Serum was collected and stored at −80°C in multiple aliquots until analyzed. Analyses of total cholesterol (Total-C), high-density lipoprotein cholesterol (HDL-C), low-density lipoprotein cholesterol (LDL-C), Apolipoproteins (Apo A-1, Apo B), glucose and insulin were performed by an accredited laboratory according to standard laboratory methods (Department of Medical Biochemistry, Oslo University Hospital, Rikshospitalet, Norway). All coefficients of variation (CVs) were less than 5%.

25-Hydroxyvitamin D (25(OH) vitamin D) (DiaSorin, Stillwater, MN, USA) was quantified by radioimmunoassay (RIA). For this analysis, all samples were measured in duplicate, with serial samples from a given individual run at the same time. According to the manufacturer, the intra- and inter-assay CVs were less than 5% and 10%, respectively.

The Homeostasis Model Assessment (HOMA) indices were calculated and performed based on fasting glucose and insulin samples. The homeostatic model assessments of β-cell function (HOMA-β) and insulin resistance index (HOMA-IR) were determined using the Oxford calculator (https://www.dtu.ox.ac.uk/homacalculator/download.php) (20).

### Body composition

Total body composition was measured by DXA as described (21). All study centers used standard imaging and positioning protocols to scan the subjects. DXA has been used increasingly as a reference method for comparison with other techniques and is considered the standard of choice for body composition measurements in clinical studies (22). DXA provides information on bone mineral content, total fat mass (FM) and total lean mass (LM), the latter a remaining fat free and bone free component. Together, these three components sum up to the patients total body mass.

In the present study, we focused on total FM (g), total LM (g) and trunk fat mass (TrFM) (g). For assessment of TrFM, a region of interest was defined with the caudal limit at the top of the iliac crest and the cephalic limit at the base of the skull. Technically, adequate DEXA scans for assessment of body composition were available for 103 patients.

For the DXA analyses, one center used Norland XR 46 system 8091 (Norland Corporation, Fort Atkinson, WI, USA) and another Hologic QDR-4500 (Hologic Inc., Bedford, MA, USA). All other centers used Lunar DPX-L, software version 4.6c (GE Healthcare, Lunar Corp., Madison, WI, USA), until 2004/2005 and thereafter Lunar Prodigy densitometer, software version 12.10 (from the same manufacturer). During the years, several centers upgraded their software version, but for this paper, all original scans were retrieved, and when necessary recalculated in the same software version. All participating centers had daily procedures for calibrations for bone and fat to avoid systematic errors between different software versions.

According to the manufacturer, the CV values for total body tissue and FM were 0.1 and 2.5%, respectively, for Lunar DPX-L instrument (regarded by the manufacturers to be similar to the Lunar Prodigy) (23, 24).

### Statistical analysis

Normally distributed data are presented as mean (±s.d.), otherwise as median (25–75 percentiles). Normality was assessed by Shapiro–Wilk test for each parameter within the randomization groups (PTX; OBS). Depending on the distribution, longitudinal changes between 0 and 5 years were assessed by paired samples *t*-test or Wilcoxon signed-rank test, as appropriate. Between-group differences (OBS vs PTX) were tested by independent samples *t*-test or Mann–Whitney *U* test, as appropriate. Parametric tests are indicated by lower-case ‘*p*’; non-parametric by upper-case ‘*P*’, in tables by *italic* font. *P*/*p* values <0.05 were considered statistically significant. The end points were analyzed according to the intention-to-treat principle.

Potential determinants for the change in LDL-C in the study period were identified by univariate analyses and assessed by linear regression analyses.

SPSS versions 23–25 were used for statistical analyses.

## Results

### Patient characteristics

Data were available from 119 patients for both the baseline and the 5-year visit. At baseline, 58 of these patients were randomized to OBS and 61 patients to PTX. In six patients randomized to OBS, parathyroidectomy was performed before the 5-year visit. According to the intention-to-treat principle, they remained in the OBS group for analyses. Demographics and basic biochemistry details are given in Table 1.

**Table 1:** Group characteristics.

Baseline characteristics	Subjects assessed OBS; PTX (*n*; *n*)	Observation (OBS)		Parathyroidectomy (PTX)	
		*n* Mean *Median*	±s.d. (25%; 75%)	*n* Mean *Median*	±s.d. (25%; 75%)
Gender (female; male)		50; 8		53; 8	
Age at inclusion (years)	58; 61	61.4	(57.4; 67.8)	61.3	(57.1; 66.5)
BMI (kg/m²)	49; 47	26.4	(23.5; 29.8)	25.9	(24.4; 29.2)
Weight (kg)	50; 53	75.1	(±12.2)	75.2	(±11.1)
PTH (pmol/L)	58; 61	9.8	(8.0; 12.6)	10.1	(7.5; 12.0)
Calcium (alb.corr.) (mmol/L)	58; 61	2.63	(±0.11)	2.64	(±0.10)

In the present cohort, baseline mean albumin adjusted calcium (Ca(alb)) was 2.63 ± 0.10 mmol/L and baseline median PTH was 9.8 (7.8; 12.4) pmol/L. In the treatment group (PTX), Ca(alb) and PTH were normalized 5 years after surgery (Ca(alb): 2.31 ± 0.10 mmol/L; PTH: 4.4 (3.6; 6.0) pmol/L). In the OBS group, a small but statistically significant decrease in Ca(alb) to 2.52 ± 0.14 mmol/L (*P* < 0.01) was noticed after 5 years of observation, but without significant changes in the PTH levels (10.5 (7.5; 12.9) pmol/L).

**Figure 1:**
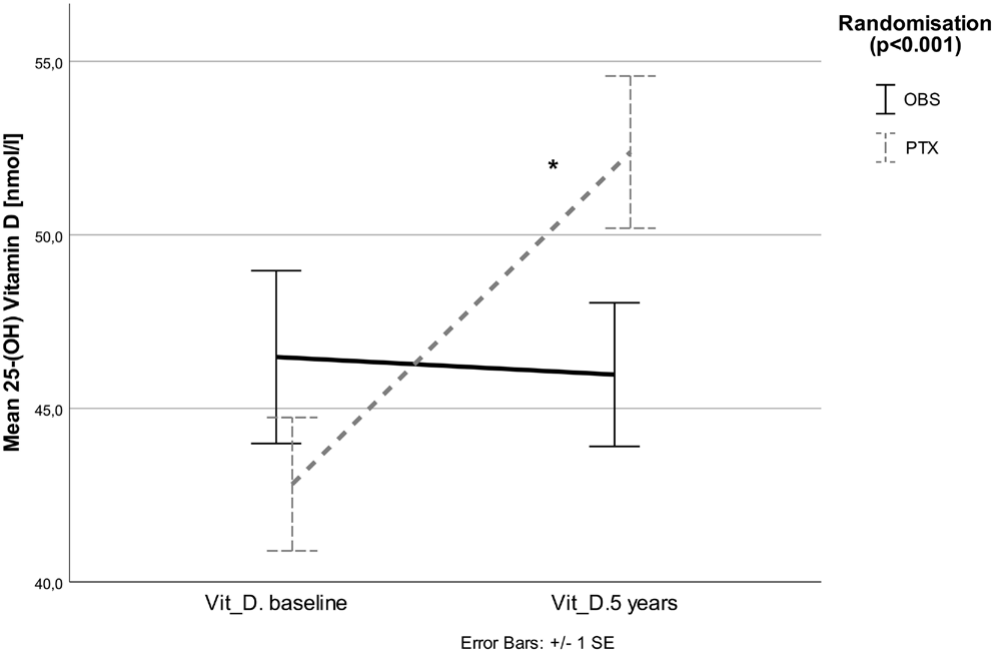
Mean 25(OH) Vitamin D increased in the PTX group (*P* < 0.001), but did not change significantly in the OBS group (*P* = 0.56). There was a treatment effect of PTX vs OBS group (*P* < 0.001).

Mean 25(OH) Vitamin D increased in the PTX group (*P* < 0.001), but did not change significantly in the OBS group (*P* = 0.56). There was a treatment effect of PTX vs OBS (*P* < 0.001, Fig. 1).

### Metabolic parameters

**Figure 2:**
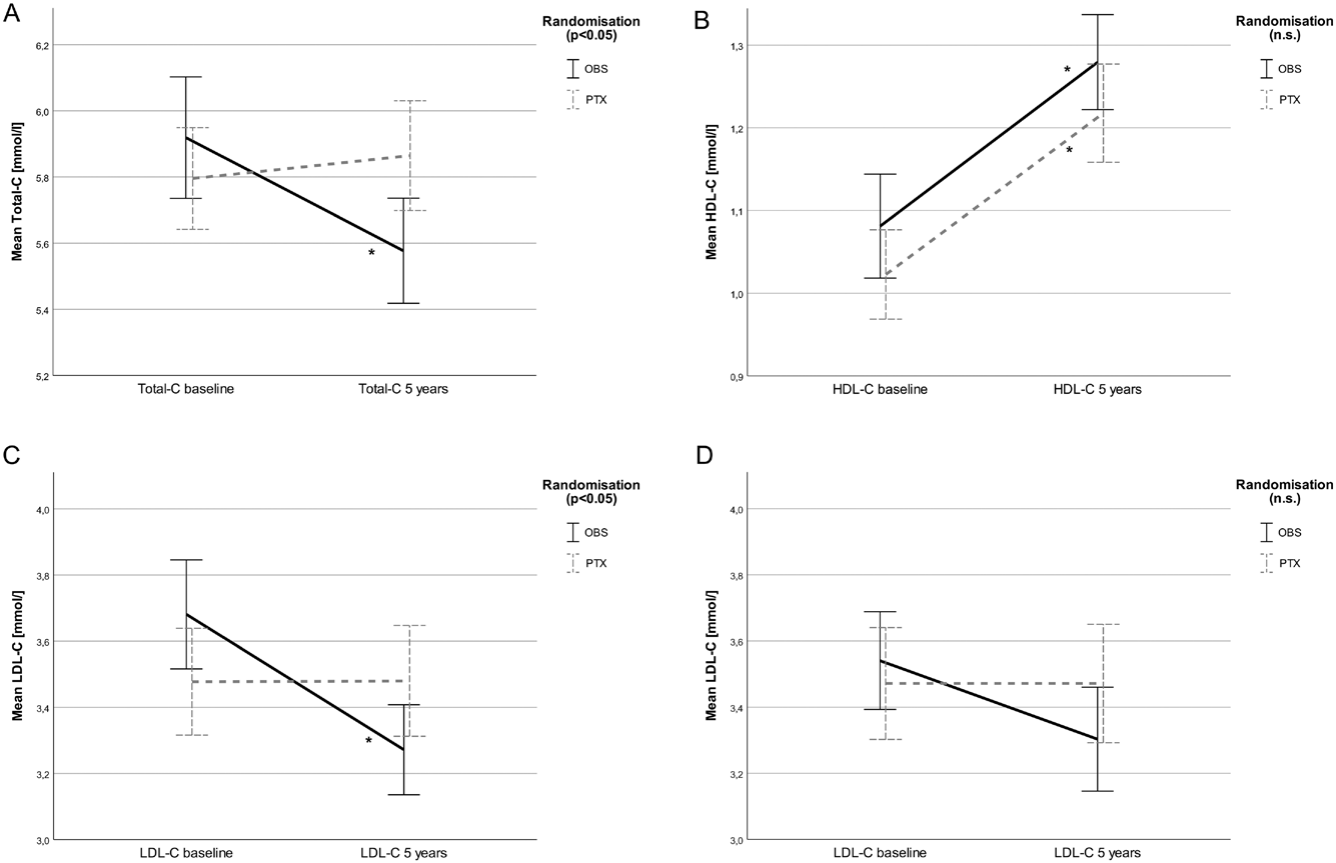
(A, B, C and D) Cholesterol levels. Mean cholesterol levels per treatment group (OBS vs PTX). OBS, observation without intervention; PTX, parathyroidectomy. Error bars: ± 1 s.e.; *significant change within group from 0 to 5 years. (A) Mean Total-C decreased in OBS (*P* = 0.037), but not in PTX. There was a difference between groups over time (*P* = 0.013 for interaction between groups). (B) Mean HDL-C increased in both groups (OBS and PTX), (*P* < 0.01), without significant difference between the groups. (C) Mean LDL-C decreased in OBS (*P* = 0.010), but not in PTX. There was a difference between groups over time (*P* = 0.026 for interaction between groups). (D) Mean LDL-C in patients not started with statins in study period. The decrease in the OBS group was not significant (*P* = 0.079), and there was no difference between groups (*P* = 0.12).

No significant changes in glucose, insulin or glucose/insulin levels (HOMA-β, HOMA-IR) were observed over 5 years in any of the two groups and no group-wise effect of treatment was observed (Table 2). Total-C and LDL-C decreased significantly in the OBS group over the observation period (*P* < 0.05; Fig. 2A and C). In the PTX group, these parameters did not change significantly. There was a significant difference in change over time between the groups, with improved total and LDL-C in the OBS group (Total-C: *P* = 0.013; LDL-C: *P* = 0.026 for interaction between groups, Fig. 2A and C). HDL-C increased in both groups (*P* < 0.01) without any significant difference between the groups (Fig. 2B).

**Table 2:** Metabolic parameters, change over time and between the two randomization groups over time (OBS vs PTX).

Variable	OBS/PTX	OBS					PTX					OBS vs PTX	Power 80%***
	*n*	Baseline	±s.d. (25%; 75%)	5 years	±s.d. (25%; 75%)	*P* 0 vs 5 years	Baseline	±s.d. (25%; 75%)	5 years	±s.d. (25%; 75%)	*P* 0 vs 5 years	P**	OBS vs PTX over time***
Glucose (mmol/L)	37/43	5.1	(4.8; 5.9)	5.4	(5.1; 5.9)	*0.238*	5.2	(4.9, 5.9)	5.4	(4.9; 5.9)	*0.967*	*0.336*	0.35
Insulin (pmol/L)	37/43	70	(46; 130)	70	(45; 113)	*0.504*	71	(55; 129)	72	(51; 129)	*0.790*	*0.780*	27
Fasting glucose ≥7 mmol/L (*n*)	7/5	4		6			5		3				n.d.
HOMA-β	30/30*	101	(73; 125)	99	(73; 125)	*0.975*	111	(89; 140)	111	(89; 140)	*0.811*	*0.858*	n.d.
HOMA-IR	30/30*	1.07	(0.75; 1.52)	1.18	(0.75; 1.52)	*0.688*	1.32	(1.01; 2.23)	1.32	(1.01; 2.23)	*0.388*	*0.921*	n.d.
Apo A-1 (g/L)	37/43	1.9	(±0.3)	1.9	(±0.3)	*0.201*	1.9	(±0.3)	1.9	(±0.3)	*0.637*	*0.530*	0.13
Apo B (g/L)	37/43	1.1	(±0.3)	1	(±0.2)	***0.007***	1.1	(±0.3)	1.1	(±0,3)	*0.935*	*0.064*	0.12
BMI (kg/m²)	49/47	26.4	(23.4; 30.0)	26.7	(23.3; 29.9)	*0.124*	25.9	(24.4; 29.2)	27.3	(24.9; 29.2)	*0.074*	*0.673*	0.82
Weight (kg)	50/53	75.1	(±12.2)	75.9	(±14.5)	0.274	75.2	(±11.1)	75.5	(±11.1)	0.660	*0.787*	2.07
DXA													
Total FM (kg)	50/53	29.2	(±9.2)	30.1	(±9.7)	0.136	28.3	(±8.8)	28.7	(±8.6)	0.562	0.462	2.17
Total LM (kg)	50/53	41.5	(37.4; 47.1)	42.3	(36.8; 48.4)	*0.508*	42.4	(40.1; 46.4)	43.0	(39.6; 46.8)	*0.905*	*0.639*	1.33
Trunk fat (kg)	44/46	14.8	(±5.4)	15.7	(±6.3)	**0.025**	14.5	(±4.3)	14.8	(±4.7)	0.469	0.228	1.55

*P*-Values: bold: <0.05; italic: non-parametric test; normal: parametric test.

*Patients with glucose ≥7.0 mmol/L at visit 0 or 5 were excluded listwise; **difference between groups for change over time; ***difference between groups that could have been detected with 80% power as measure for potential type 2 error.

Six of the patients with available samples for cholesterol analyses started with cholesterol-lowering pharmacological treatment (statins) during the study period, five in the OBS group, but only one in the PTX group. When statin starters were excluded from the analyses, the difference in LDL change between OBS and PTX was no longer significant (*P* = 0.12, Fig. 2D).

In order to identify potential determinants for the change in LDL-C, we performed a linear regression analysis. In a univariate model, we identified the initiation statin therapy during the course of the study (*P* = 0.005), baseline LDL-C (*P* < 0.001) and randomization (*P* = 0.054) to correlate (or trend) with change in LDL-C and thereby as potential determinants for the change in LDL-C (Table 3). When baseline LDL-C and statin initiation were included in the linear regression model, randomization did not contribute significantly to the model for determination of LDL-C change (*P* = 0.18). Thus, parathyroidectomy was not a significant factor for change in LDL-C when corrected for statin use and baseline LDL-C.

**Table 3:** ****Linear regression analyses with change in LDL-C as dependent variable.

Predictors	Unadjusted estimates					Adjusted estimates (model summary: *R*^2^ = 0.294)			
	*R*^2^	Un-standardized β	Standardized β	95% CI	*P*	Un-standardized β	Standardized β	95% CI	*P*
Randomization (OBS/PTX)	*0.047*	0.415	0.216	−0.004; 0.437	0.054	0.259	0.135	−0.004; 0.437	0.176
LDL-C at baseline	*0.235*	−0.448	−0.485	−0.682; −0.288	<0.001	−0.396	−0.429	−0.627; −0.232	<0.001
Statins started in study period	*0.096*	−1.131	−0.310	−0.525; −0.096	0.005	−0.664	−0.182	−0.383; 0.019	0.075

Apo A-1 did not change in any of the two randomization groups during the study period. There was a small, but significant decrease in Apo B in the OBS group (*P* = 0.007). In the PTX group, Apo B did not change (*P* = 0.94). There was a trend toward a between-group difference (*P* = 0.064 for interaction between groups) in favor of observation.

### Change in fat distribution in DXA measured fat and lean mass

**Figure 3:**
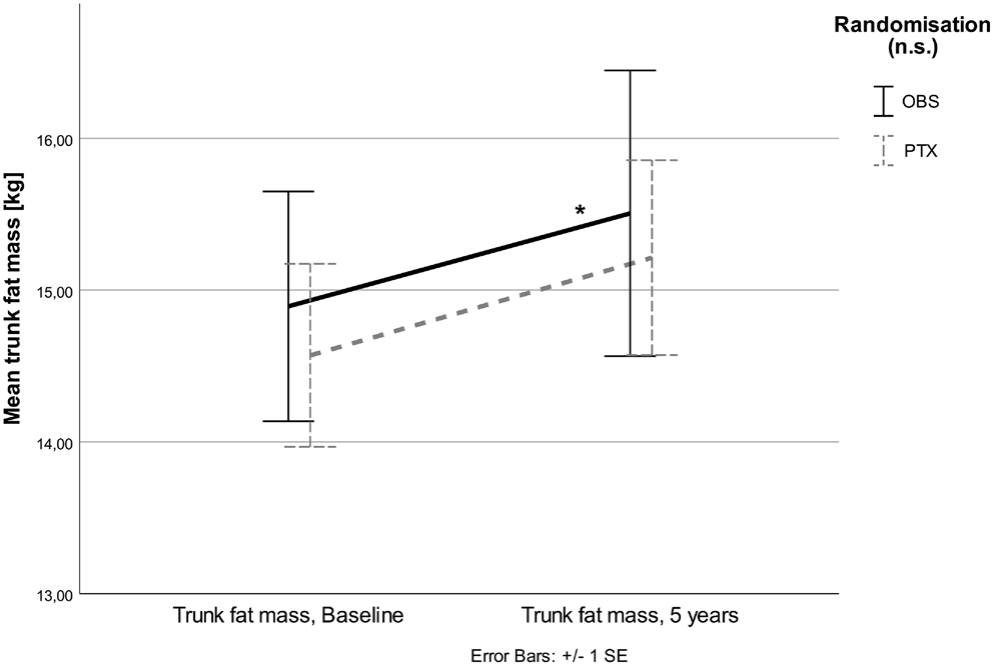
Mean TrFM increased in OBS group (*P = 0.025), but not in PTX. Nevertheless, no significant interaction between randomization groups (*P* = 0.228) was detected.

BMI and total body weight did not change significantly in any of the two groups during 5 years after randomization. In the OBS group, total FM and LM did not change after 5 years, but TrFM increased (*P* = 0.02, Fig. 3).

No significant changes were seen in the PTX group for these parameters. However, no significant treatment effects for any of the analyzed body composition compartments were observed over the five years of observation.

## Discussion

In this randomized study of patients with mild PHPT followed for 5 years, we found a decrease in Total-C and LDL-C in the observation group, despite a small, but significant increase in TrFM, while these parameters remained unchanged in the PTX group, resulting in a significant treatment effect for Total-C and LDL-C in favor of observation. As expected, a significant positive treatment effect of PTX on 25(OH) vitamin D levels was demonstrated, as compared to observation.

Mild PHPT has been associated with increased frequency of dyslipidemia, overweight, impaired glucose tolerance, diabetes mellitus and diseases in the cardiovascular system in numerous studies (7, 11, 25, 26). Studies addressing the effect of surgery on cardiovascular risk factors have reported conflicting results (8, 11, 15, 25). These studies were non-randomized case–control studies (11), had no observational non-PTX control group and were of relatively short duration (8, 15, 25). Even in an early study including many patients with overt PHPT, a positive effect on renal function and arterial hypertension was not detected (27, 28).

The present results 5 years after randomization did not indicate the hypothesized positive effect of PTX on cardiovascular risk markers in mild PHPT, but rather a potential positive effect of observation on Total-C and LDL-C. A similar decrease in LDL-C has been described previously (11), but in that study a decrease was observed both in OBS and PTX patients. As in our study, an increase in HDL-C was described in both OBS and PTX (11). The regression analysis in the present study demonstrates that the decline in LDL-C was driven by patients with high LDL-C at baseline and the uneven distribution of patients starting with statins within the study period.

There was no significant change in total FM in any of the groups. However, TrFM increased in the OBS group. This is a potentially negative metabolic change and seems to be in contrast to the changes observed on cholesterol parameters. A possible explanation for these apparently opposing observations may be the effect of lipid-lowering drugs, as discussed earlier. This assumption is supported by findings from the Norwegian population-based ‘Tromsø study’, where a substantial decrease in mean Total-C levels in the general population was observed in the same time period and age groups as in our present study (29). Further, the open design of the present study with no intervention in mild PHPT might have encouraged patients in the OBS group toward a healthier lifestyle and better compliance to cardiovascular risk lowering drugs such as lipid-lowering medication.

As for lipid metabolism, there is a large amount of observational data indicating disturbances in glucose metabolism associated with PHPT (28, 30, 31). Nevertheless, evidence for the beneficial effect of PTX in mild PHPT is scarce (13). In single arm studies following patients after PTX, a trend toward an increase of insulin resistance (HOMA-IR and QUICKI) was found in a single recent study (32), while another study found some improvement in glucose metabolism in non-diabetic patients (25). The present randomized, controlled study did not indicate a longitudinal change or between-group difference in the markers of the metabolic syndrome.

The main strengths of the present study are the randomized design and the long-term follow-up. The combined analyses of body composition and lipid profile comparing parathyroidectomy with medical observation is unique in mild PHPT. However, certain limitations have to be addressed. As some patients did not have technically satisfactory scans to assess body composition, the number of patients with DXA scans was lower than that in the previous publications from the SIPH study assessing sceletal aspects of mild PHPT (19). For this study, only a subgroup of patients could be analyzed due to lack of follow-up DXA scans and frozen blood samples in some of the patients who completed the 5-year visit in this multi-national investigator initiated study. As a consequence, the difference in change of cholesterol variables between OBS and PTX differs from previously published results after 2 years (9). In these previous analyses, no significant difference between the two randomization groups was found. Unfortunately, the analyses of cholesterol and other metabolic parameters were limited to patients with available frozen blood samples. Although there was a large overlap between the 2- and 5-year cohorts, the present cohort was smaller than in the previously published 2-year results. In analyses restricted to patients with complete biochemical datasets at all three time points (0, 2 and 5 years; Total-C and LDL-C), we observed a difference between groups already after 2 years (*P* < 0.05). The power to detect minor changes in parameters assessed in this study was limited (Table 2). Further, there were more patients starting with statins in the OBS group. Thus, we assume that the current results with an improvement of Total-C and LDL-C in the OBS group were caused by a combination of more OBS patients starting with statins, as discussed earlier, and a type 1 error caused by selection bias of patients included in the present cohort.

Taken together, we observed a positive effect of observation on LDL-C and Total-C in contrast to the hypothesized positive effect of PTX in patients with PHPT. However, differences in treatment with statins during the study and a potential bias in the analyzed cohort may have been confounding factors.

In conclusion, our results do not support that parathyroidectomy has positive metabolic effects on lipid and glucose metabolism and should not be a separate indication for surgery to modify cardiovascular risk factors in mild PHPT.

## Declaration of interest

The authors declare that there is no conflict of interest that could be perceived as prejudicing the impartiality of the research reported.

## Funding

The SIPH study has received support from the Norwegian Research Council and from Swedish Government grants under the ALF-agreement (ALF VGR-71160), The Göteborg Medical Society and unrestricted research grants from Novo Nordisk Norway AS, Biovitrium AB, Sweden and Amgen AB, Sweden.
